# Supplementary figures and images for: Automated, phylogeny-based genotype delimitation of the Hepatitis Viruses HBV and HCV
Source: PeerJ. 2019 Oct 25;7:e7754. doi: 10.7717/peerj.7754 (PMC6816385; doi:10.7717/peerj.7754)

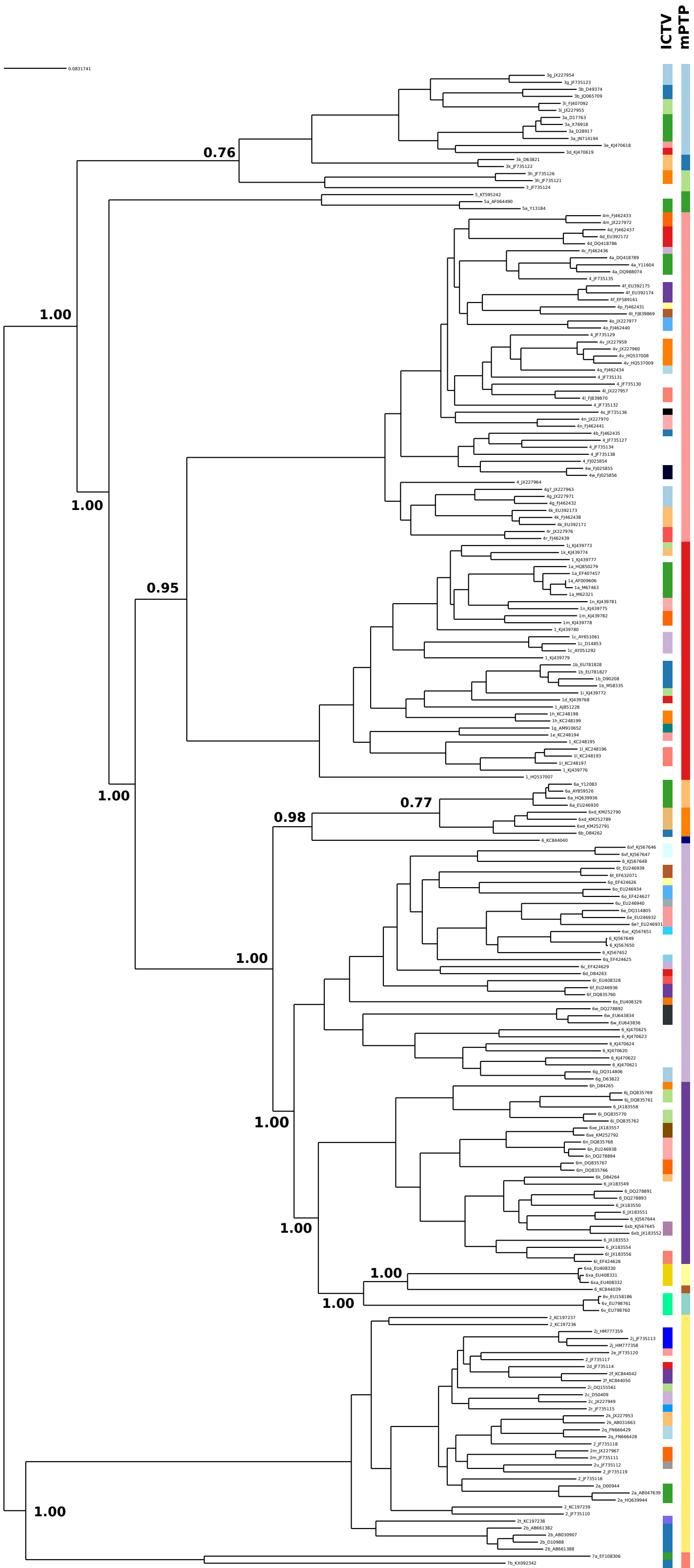

Supplement: Figure S1 — Comparison of the subtypes of HCV, indicated by the first colored column, to the mPTP delimited clusters, indicated by the second colored column. The numbers indicate the support for a particular node being a speciation node (support <0.5 not shown, see also Fig. 1 in main text and Fig. S2) obtained by the MCMC sampling under the mPTP model. [file peerj-07-7754-s001.pdf]

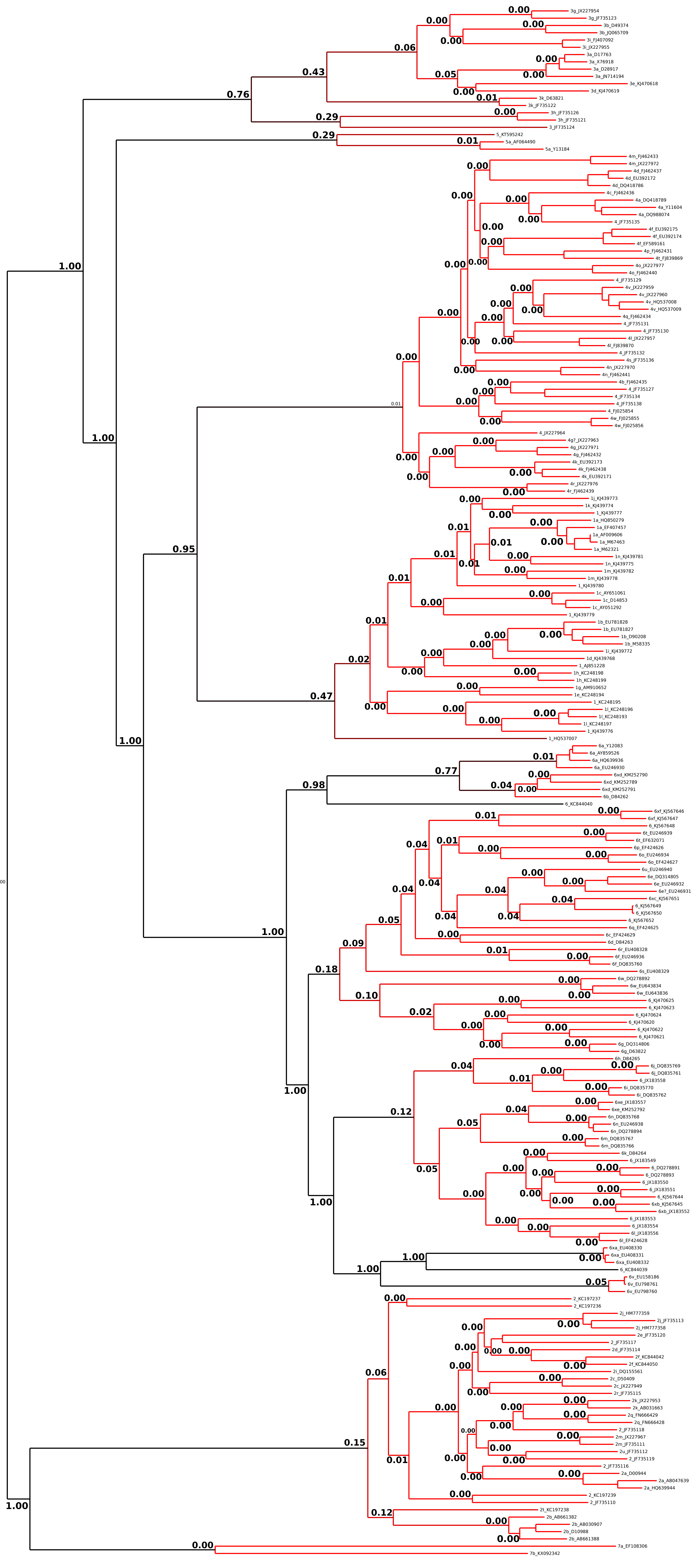

Supplement: Figure S2 — Raw output of MCMC sampling analysis for the HCV dataset under the mPTP model showing the support of all nodes being speciation nodes. The color of the branches of the tree range from black to red in proportion to the MCMC support for each branch being part of the speciation processes (i.e., black branches were always sampled as speciation branches and red branches were always sampled as coalescent branches). [file peerj-07-7754-s002.pdf]

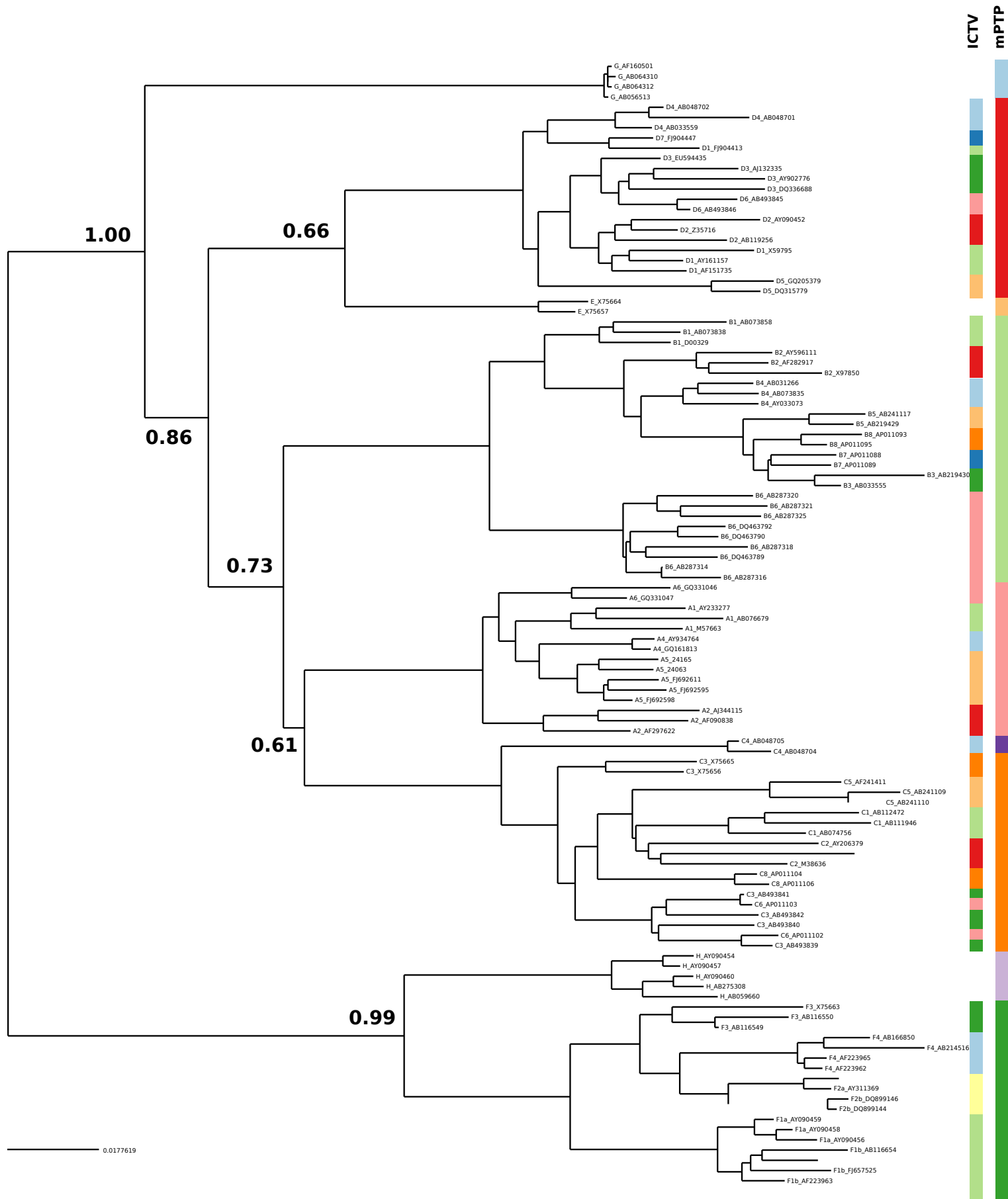

Supplement: Figure S3 — Comparison of the ssubgenotype of HBV, indicated by the first colored column, to the mPTP delimited clusters, indicated by the second colored column. The numbers indicate the support for a particular node being a speciation node (support <0.5 not shown, see also Fig. 1 in main text and Fig. S2) obtained by the MCMC sampling under the mPTP model. [file peerj-07-7754-s003.pdf]

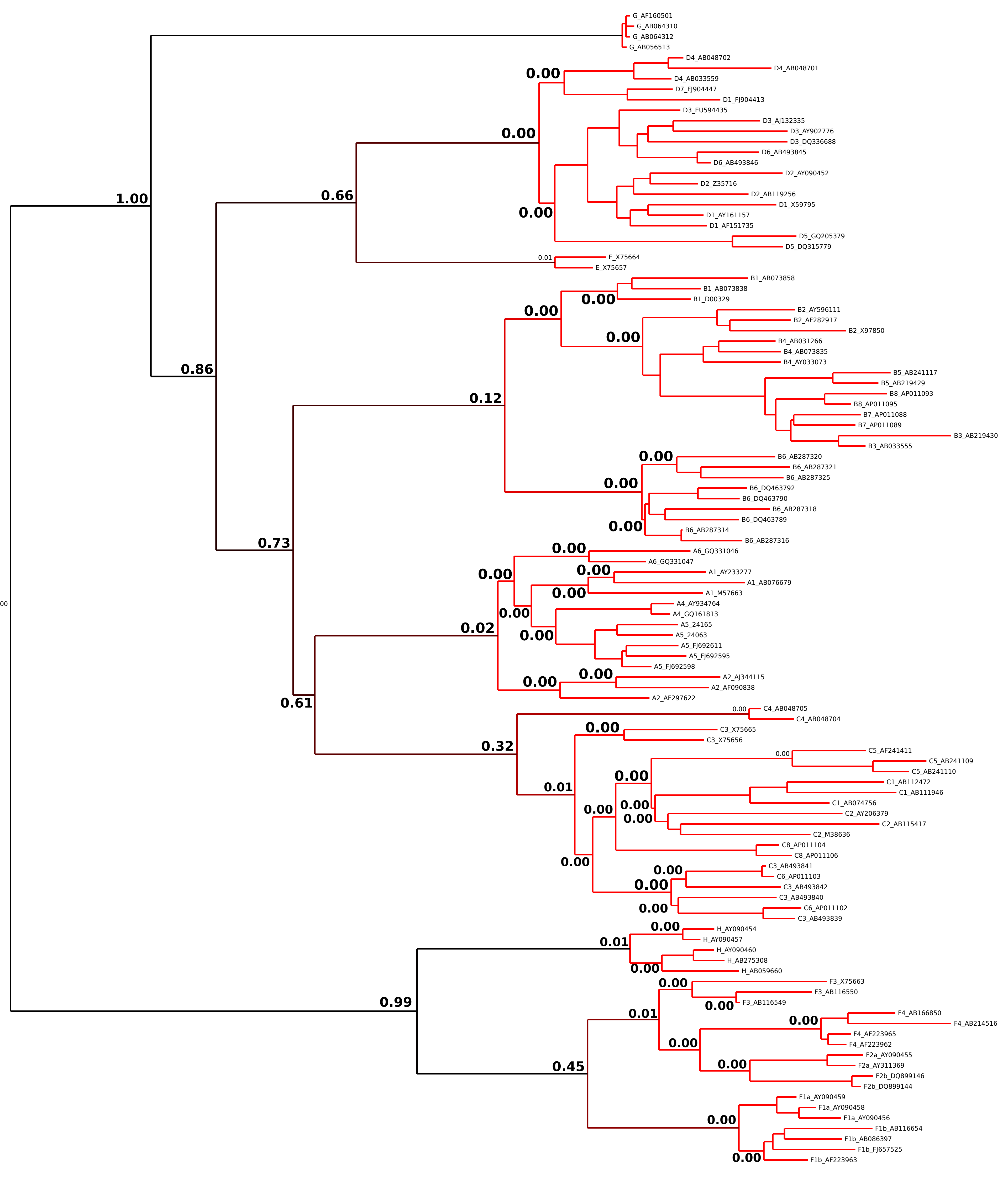

Supplement: Figure S4 — Raw output of MCMC sampling analysis for the HBV dataset under the mPTP model showing the support of all nodes being speciation nodes. The color of the branches of the tree range from black to red in proportion to the MCMC support for each branch being part of the speciation processes (i.e., black branches were always sampled as speciation branches and red branches were always sampled as coalescent branches). [file peerj-07-7754-s004.pdf]
